# HaemSTAR—Growing Experience From a National, Trainee-led Research Network

**DOI:** 10.1097/HS9.0000000000000766

**Published:** 2022-08-10

**Authors:** Lydia Wilson, Andrew J. Doyle, Emily Millen, Tom Bull, Tina Biss, Dan Hart, Gillian Lowe, Cheng-Hock Toh, Richard J. Buka, Phillip L. R. Nicolson

**Affiliations:** 1 Bart’s Health NHS Trust, London, United Kingdom; 2 Thrombosis and Haemophilia Centre, Guy's and St Thomas' NHS Foundation Trust, London, United Kingdom; 3 Clinical Haematology, Nottingham University Hospitals NHS Trust, Nottingham, United Kingdom; 4 Haematology, West Suffolk NHS Foundation Trust, Bury St Edmunds, United Kingdom; 5 Department of Haematology, Newcastle upon Tyne, United Kingdom; 6 Department of Immunobiology, Barts and The London School of Medicine and Dentistry, Blizard Institute, Queen Mary University of London, United Kingdom; 7 Comprehensive Care Haemophilia Centre, University Hospitals Birmingham NHS Foundation Trust, Birmingham, United Kingdom; 8 Department of Haematology, Liverpool University Hospitals NHS Foundation Trust and University of Liverpool, United Kingdom; 9 Institute of Cardiovascular Sciences, College of Medical and Dental Sciences, University of Birmingham, United Kingdom

Participation in clinical research results in improved outcomes, not only for participants but also for other patients treated at centers with research engagement.^1–3^ Furthermore, while malignant hematology is a hotbed of research, nonmalignant hematology is relatively underserved aside from a few exceptions.^4^

In an attempt to improve clinical research in nonmalignant hematology in the United Kingdom, the National Institute for Health Research Haematology Clinical Research Network set up Haematology Speciality Training, Audit, and Research (HaemSTAR) in 2017, an independent network of UK clinical hematology trainees interested in nonmalignant hematology. The organization currently consists of a committee of 5 with regional representatives in all 21 educational deaneries within the UK acting as “hubs.” Trainee collaborators are identified and supported by these regional representatives.^5^ Five years on from its inception, we review the progress made by HaemSTAR.

Publications by the network were identified through internal records and data were extracted from manuscripts. Site involvement was defined by author affiliations. Participating sites were defined as “academic” and “non-academic” according to medical school affiliation and/or membership of the University Hospital Association.^1^

As of December 31, 2021, HaemSTAR has contributed to 11 published manuscripts and 6 abstracts involving 9405 patients, which are summarized in Table 1. HaemSTAR either led or assisted with projects, with the former recently adopting a collaborative-authorship model to recognize the contribution of trainee collaborators. The median number of authors or citable collaborators in these projects is 10 (range, 6–136) and the median number of sites involved in HaemSTAR publications is 15 (range, 6–80). Publishing journals had an impact factor greater than 1.0, with the median of 2.63 (range, 1.05–91.24). Figure 1 shows the wide geographical area HaemSTAR covers and demonstrates the involvement of a large spectrum of acute hospital trusts. In total, HaemSTAR has had research contributions from 97 individual sites in 65 of 223 (26%) National Health Service (NHS) hospital trusts with 39 (40%) being major teaching hospitals.^21^

**Table 1:** HaemSTAR Published Papers and Abstracts

Title	Year	Authors/Collaborators	Study Participants	Type of Study	Open Access?	HaemSTAR Involvement
A HaemSTAR is born; a trainee-led, UK-wide research network in haematology^5^	2019	6	NA	Letter	Yes	Fully HaemSTAR delivered
An international survey of clinicians regarding their management of venous thromboembolism following the initial 3-6 months of anticoagulation^6^	2020	7 (HaemSTAR acknowledged)	351	Survey	No	Supported survey dissemination
A single 1 g/kg dose of intravenous immunoglobulin is a safe and effective treatment for immune thrombocytopenia; results of the first HaemSTAR ‘Flash-Mob’ retrospective study incorporating 961 patients^7^	2021	136	978	Audit	Yes	Fully HaemSTAR delivered
An assessment of the management of anaemia in acute care settings in the United Kingdom: the value of a collaborative approach^8^	2021	3 (plus 21 HaemSTAR collaborators)	828	Audit	No	Fully HaemSTAR delivered
Mycophenolate mofetil for first-line treatment of immune thrombocytopenia^9^	2021	13 (HaemSTAR acknowledged)	120	RCT	No	Supported recruitment—provided 31% of sites
The negative impact of the COVID-19 pandemic on UK haematology registrars’ well-being and training: results of a UK nationwide survey^10^	2021	4 (HaemSTAR acknowledged)	89	Survey	Yes	Fully HaemSTAR delivered
The clinical course of COVID-19 in pregnant versus non-pregnant women requiring hospitalisation: results from the multicentre UK CA-COVID-19 study^11^	2021	17 (HaemSTAR acknowledged)	36	Retrospective observational	Yes	Supported site registration
Clinical outcomes and the impact of prior oral anticoagulant use in patients with coronavirus disease 2019 admitted to hospitals in the UK — a multicentre observational study^12^	2022	30 (HaemSTAR acknowledged)	5883	Retrospective observational	Yes	Supported site registration
Diagnostic uncertainty presented barriers to the timely management of acute thrombotic thrombocytopenic purpura in the United Kingdom between 2014 and 2019^13^	2022	10 plus “HaemSTAR collaborators”	148	Audit	Yes	Fully HaemSTAR delivered
Platelet transfusion and anticoagulation in hematological cancer-associated thrombosis and thrombocytopenia: the CAVEaT multicenter prospective cohort^14^	2022	7 plus “HaemSTAR network”	105	Prospective cohort	Yes	Supported recruitment—provided 70% of sites
A real-world study of immune thrombocytopenia management during the COVID-19 pandemic in the UK^15^	2022	5	24	Prospective observational	Yes	Supported site recruitment
Published abstracts						
Real world effectiveness of thrombopoietin receptor agonists (TRAs) in the management of immune thrombocytopenia (ITP) in the UK: results from a post hoc analysis of the TRAIT study^16^	2019	19	267	Retrospective observational	Yes	Supported recruitment—provided 39% of sites
Real world use of thrombopoietin-receptor agonists in the management of immune thrombocytopenia in the United Kingdom: results from the TRAIT study^17^						
Outcomes of 99 patients with myeloproliferative neoplasm (MPN) associated splanchnic vein prognosis (SVP): first report from UK MASCOT registry^18^	2020	8 plus “HaemSTAR network”	99	Registry study	Yes	Supported recruitment
BSH 2021 OR-026: Factors influencing time from initial presentation to start of plasma exchange (PEX) in patients with acute thrombotic thrombocytopenic purpura (TTP)^19^	2021	10 plus “HaemSTAR collaborators”	148	Audit	Yes	HaemSTAR delivered all

Where abstracts were followed by full publications, only the full publication is included.

HaemSTAR = Haematology Speciality Training, Audit, and Research; RCT = randomized controlled trial.

**Figure 1.**
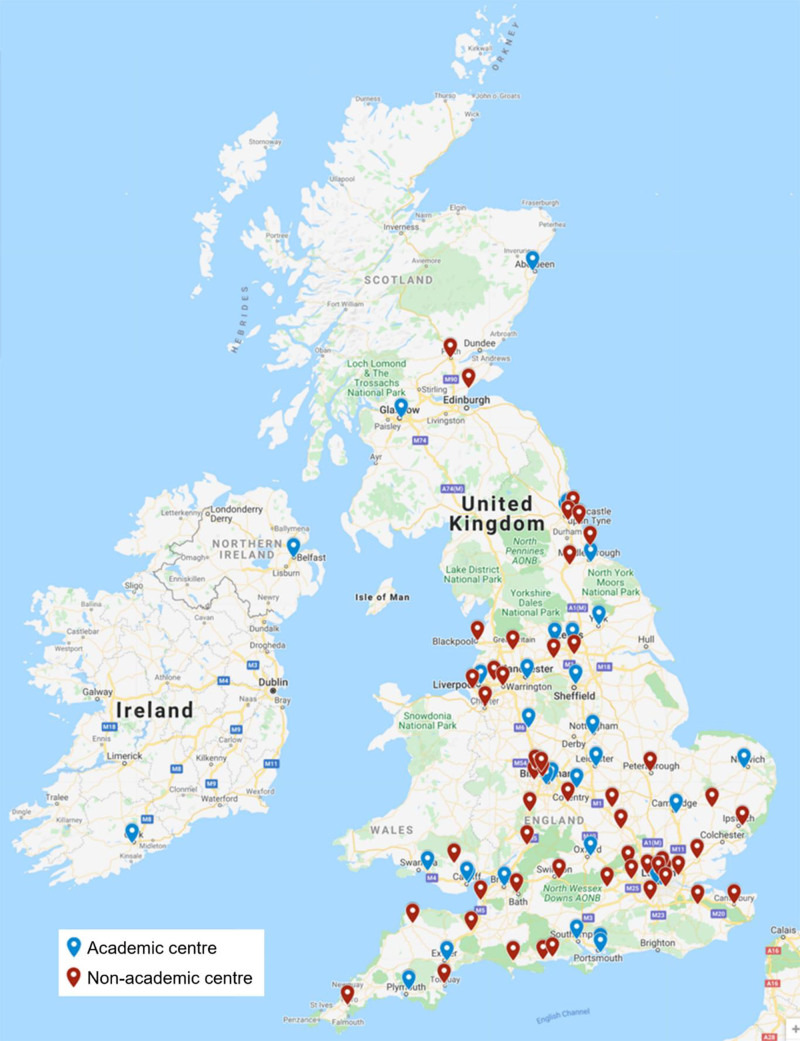
**Centers in United Kingdom and Ireland involved in HaemSTAR.** ^20^ HaemSTAR = Haematology Speciality Training, Audit, and Research.

HaemSTAR began by supporting existing projects having been approached by principal investigators (PIs) to improve recruitment. For example, First Line treatment pathways for newly diagnosed Immune Thrombocytopenia (FLIGHT), a randomized controlled trial of mycophenolate mofetil for first-line treatment of immune thrombocytopenic purpura (ITP), engaged the support of the network subsequently, enrolling a further 13 sites (31% of total), recruiting 43 of 120 patients (36%) (Table 1), and contributing £8964 research income.^9^

In 2018, HaemSTAR launched the first of its flagship “flash-mob” projects, snap-shot audits designed to enlist the help of many people over a very short period.^7^ This audit is the largest ever reported patient cohort of IVIg in ITP and found no difference between a one-off dose of 1 g/kg and 2 doses of 1 g/kg on consecutive days. Extrapolated, this supports annual cost-savings of over £1 million per year in England.^7^ The second flash-mob audit looked at care and outcomes of patients with thrombotic thrombocytopenic purpura and has generated data that has been used to support the commissioning of specialist centers across the United Kingdom.^14^ The third flash-mob project is in late development and will audit the real-world use of reversal agents for direct oral anticoagulants. This project has attracted nearly £100,000 funding from industry in the form of academic grants and administration support.

During the COVID-19 pandemic, HaemSTAR mobilized quickly to understand complications of the disease and further showed its value with work in collaboration with Public Health England (PHE). In the Coagulopathy Associated with COVID-19 (CA-COVID) study, multiple aspects of hemostasis and thrombosis in patients admitted with COVID-19 were investigated with 14 of 28 (50%) centers recruited through involvement of HaemSTAR collaborators. The study recruited over 5000 patients and has resulted in 2 publications.^11,12^ The PI described “the study as successful because of the great support from HaemSTAR.” In 2021, HaemSTAR collected vital patient-level data for PHE to ascertain the incidence of vaccine-induced immune-mediated thrombotic thrombocytopenia confirming diagnostic criteria and incidence at a national level (submitted for publication).

In its short existence, HaemSTAR has progressively increased its publication and presentation output, and given research experience to trainee collaborators. The “hub-and-spoke” model enables swift and thorough dissemination of research opportunities, questionnaires, and research findings. The flash-mob model allows clinical questions to be answered quickly using a core research team who organize regional representatives and data collection. This is important when collaborators lead busy clinical lives with little dedicated research experience.

UK research is generally centered around university teaching hospitals, which fails to include many patients with better clinical outcomes at research-active centers.^2,3^ HaemSTAR’s involvement of a quarter of hospital sites in the United Kingdom is promising but leaves a large number of hospitals yet to be involved. While HaemSTAR’s reach to smaller hospitals is a strength, there remains bias towards involvement of academic centers potentially due, at least in part, to the presence of more trainees.

So far, HaemSTAR has delivered mainly observational studies and audits. Going forward, the network should aim to deliver prospective, interventional studies that produce gold-standard evidence, answering important questions with direct clinical impact. In so doing, this will provide vital exposure and training in research methods to PIs of the near-future. Clearly, there are challenges including the acquisition of funding, trainees’ time commitment, and continuity. Funding studies is challenging but HaemSTAR’s track-record and unique reach within the United Kingdom is a major strength, and there is growing momentum. The most important challenge, however, should be seen as retaining independence. As HaemSTAR continues to be efficient, dynamic, and productive, we are keen to maintain its autonomy to support trainees’ clinical research experience. We recognize that HaemSTAR collaborators’ time is a precious, finite resource. The committee must ensure it is deployed judiciously and continues to generate data and outcomes that improve patient care. Finally, collaborators must be individually recognized.

HaemSTAR, as a trainee-led organization for trainees, has widened access to clinical research, provided training and produced high-quality, meaningful data in a cost-effective manner over the past 5 years. Going forwards, it should build upon this to adopt new study types and continue to engage with more sites nationally.

## AUTHOR CONTRIBUTIONS

LW attests that all listed authors meet authorship criteria and that no others meeting the criteria have been omitted. All authors were involved in the planning, conduct, and reporting in this submission.

## DISCLOSURES

The authors have no conflicts of interest to disclose.
